# Metazoan tsRNAs: Biogenesis, Evolution and Regulatory Functions

**DOI:** 10.3390/ncrna5010018

**Published:** 2019-02-18

**Authors:** Shengqian Dou, Yirong Wang, Jian Lu

**Affiliations:** State Key Laboratory of Protein and Plant Gene Research, Center for Bioinformatics, School of Life Sciences, Peking University, Beijing 100871, China; sqdou@pku.edu.cn (S.D.); yr.wang@pku.edu.cn (Y.W.)

**Keywords:** tRNA-derived small RNAs, tsRNAs, translational regulation, stress response, Argonaute

## Abstract

Transfer RNA-derived small RNAs (tsRNAs) are an emerging class of regulatory non-coding RNAs that play important roles in post-transcriptional regulation across a variety of biological processes. Here, we review the recent advances in tsRNA biogenesis and regulatory functions from the perspectives of functional and evolutionary genomics, with a focus on the tsRNA biology of *Drosophila*. We first summarize our current understanding of the biogenesis mechanisms of different categories of tsRNAs that are generated under physiological or stressed conditions. Next, we review the conservation patterns of tsRNAs in all domains of life, with an emphasis on the conservation of tsRNAs between two *Drosophila* species. Then, we elaborate the currently known regulatory functions of tsRNAs in mRNA translation that are independent of, or dependent on, Argonaute (AGO) proteins. We also highlight some issues related to the fundamental biology of tsRNAs that deserve further study.

## 1. Introduction

Transfer RNAs (tRNAs) are a class of non-coding RNAs that are crucial for protein synthesis in both prokaryotic and eukaryotic cells. Metazoan small non-coding RNAs, such as microRNAs (miRNAs), small interfering RNAs (siRNAs) and PIWI-interacting RNAs (piRNAs), regulate gene expression via the RNA interference (RNAi) mechanism [1,2]. With the advent of deep-sequencing, a large number of tRNA-derived small RNAs (tsRNAs, also called “tRNA-derived fragments” or tRFs) have been discovered in various species ranging from prokaryotes to eukaryotes [3,4,5,6,7,8,9], which has considerably expanded the small RNA repertoire. Many studies suggest that tsRNAs are not random degradation products of tRNAs but play important roles in many cellular activities such as DNA damage response [10], cell proliferation and cancer progression [11,12,13,14], transposon silencing [15,16], sperm maturation [6,17], and epigenetic inheritance of metabolism-alteration induced traits [18]. Collectively, these studies have significantly advanced our understanding of the biogenesis and the molecular functions of tsRNAs [5,19,20,21,22].

Here, we review the recent advances in tsRNA biogenesis and regulatory functions from the perspectives of functional and evolutionary genomics, with a focus on the tsRNA biology of *Drosophila* as revealed by our recent study [23].

## 2. The Biogenesis of tsRNAs under Physiological and Stressed Conditions

**Figure 1:**
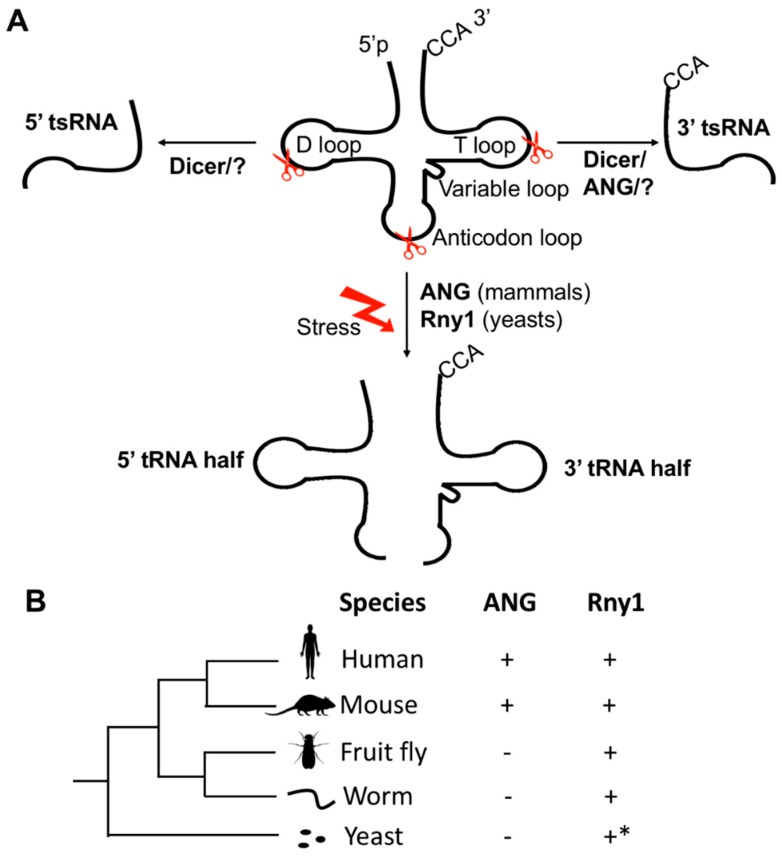
The biogenesis of tRNA-derived small RNAs (tsRNAs) from mature transfer RNAs (tRNAs). (**A**) Processing and classification of tsRNAs derived from mature tRNAs. The cleavage of an RNase at the D-loop or T-loop of a tRNA can generate a 5′ or 3′ tsRNA, respectively. The 5′ tsRNA could be generated by DCR, while 3′ tsRNA could be produced by DCR or ANG. Other unknown RNases (?) might also participate in tsRNA generation. The tRNA halves are cleaved by Rny1 and angiogenin (ANG) in yeasts and mammals, respectively. (**B**) The conservation patterns of Rny1 and ANG in eukaryotes. Among the five representative species, ANG is only present in humans and mice. Although the homologous sequence of Rny1 can be found in all the five species, currently Rny1 is only demonstrated to be involved in the biogenesis of tRNA halves in yeasts.

The cloverleaf structure of a tRNA typically contains a D-loop, an anticodon loop, a variable loop, a T-loop, and an amino acid acceptor stem [24] (Figure 1A). Different endonucleases can cleave tRNAs at specific sites, generating tsRNAs of different categories [5,7,19,20,21,22,25]. tsRNAs can also be derived from pre-tRNAs [26]; here we will focus on those derived from mature tRNAs. Based on the cleavage sites in tRNAs, tsRNAs derived from mature tRNAs can be broadly divided into two categories (Figure 1A).

The first category is produced by specific cleavage in the anticodon loop of a mature tRNA to generate a “tRNA half” which is ~35 nt in length [27,28]. The production of tRNA halves is inducible upon various cellular stresses, such as oxidative stress [29,30], arsenite, heat shock, ultraviolet irradiation [31], or virus infection [32]; therefore, tRNA halves are also called tRNA-derived stress-induced RNAs (tiRNAs) [31,33,34]. In yeasts and mammals, the tRNA-halves are cleaved by Rny1 [35] and angiogenin (ANG) [31], respectively. Rny1 is a member of the RNase T2 family that is conserved in eukaryotic genomes [36]. Interestingly, although the deletion of Rny1 in yeast can be rescued by human RNASET2 (the ortholog of Rny1), no evidence has been found that human RNASET2 participates in the biogenesis of tsRNAs in the stressed human cells [35] (Figure 1B). ANG, a vertebrate-specific member of the RNase A family, is only present in certain vertebrates, and its ortholog cannot be found in yeasts or invertebrates such as *Drosophila* and worms [37] (Figure 1B). Therefore, the biogenesis mechanisms of tRNA-halves have evolved in eukaryotes.

The secondary category of tsRNAs are cleaved in the D-loop or T-loop of a tRNA to produce short 5′-tsRNAs or 3′-tsRNAs, which are ~15–32 nt in length [27,28]. It is reported that ANG is involved in the production of 3′-tsRNAs [38]. Some studies suggest the biogenesis of this category of tsRNAs might be dependent on Dicer [9,10,39], however, others suggest Dicer is not essential for biogenesis of the tsRNAs [27,28,38,40]. These discrepant observations might be caused by the fact that tsRNAs of this category are generated by diverse mechanisms that are not evolutionarily conserved, although we cannot exclude the possibility that these discrepant observations might be caused by variations in experimental approaches and platforms used by different studies. Overall, the mechanism by which this category of tsRNAs is cleaved is yet not well understood.

Here, we collectively called the two categories of tRNA-derived small RNAs tsRNAs, unless specifically noted. The majority of tsRNAs are located in the 5′-end of tRNAs in mammals [22,27,28] and *Drosophila* [23,27]. Also, tsRNAs are preferentially generated from particular tRNAs [27]. For example, in *Drosophila*, the tsRNAs are significantly enriched in tRNA^Gly^, tRNA^Glu^, tRNA^Lys^, and tRNA^Asp^ [23]. Of note, the observation that the majority of tsRNAs are derived from the 5′-end of tRNAs and from specific tRNAs might also be caused by sequencing bias of specific tRNA species. For instance, the post-transcriptional modifications on certain tRNAs can interfere with reverse transcription and sequencing, especially for tsRNAs or mature tRNAs [41,42]. Interestingly, the temporal and spatial expression patterns of tsRNAs are regulated during animal development [5,27,28,43,44]. By examining 495 *Drosophila* small RNA libraries that span 21 cell lines or developmental stages, we found the abundance ratio of tsRNAs to miRNAs varied widely across samples, with the ratio highest in pupae and lowest in embryos and adult heads [23]. Moreover, the expression levels of tsRNAs and their binding of the Argonaute (AGO) proteins are age-dependent in *Drosophila* [43]. In addition, some short tsRNAs could also be induced by stress; for example, a class of 19-nt tsRNAs is produced in phosphate-starved roots of *Arabidopsis* [45].

## 3. Conservation of tsRNAs in the Tree of Life

The RNAi-based small RNA pathways are not evolutionarily conserved in eukaryotes. The siRNA pathway is the most ancient form; the miRNA pathways originated independently in plants and animals, and the piRNA pathway is primarily present in animal germlines [46]. By contrast, high-throughput sequencing results indicate that tsRNAs exist in all the domains of life, with some tsRNA sequences nearly identical between humans and bacteria [27]. The conservation patterns of tsRNAs were more pronounced when we focused on two *Drosophila* species, *D. melanogaster* and *D. virilis*, which diverged more than 60 million years ago [47]. We previously found that 83.4% of the tsRNA species detected in *D. virilis* have the identical sequences detected in the small RNA sequencing libraries of *D. melanogaster*, and the abundance of the 5′ tsRNAs was significantly correlated in these two species [23]. Overall, these results suggest that tsRNAs have a very ancient origin, and the sequences and expression levels of tsRNA can be highly conserved. It will be interesting to investigate in the future whether the conserved tsRNAs have conserved target genes that are maintained due to functional constraints during evolution.

## 4. tsRNA-Mediated AGO-Independent Translational Regulation

Despite the growing knowledge of tsRNAs, the underlying molecular mechanisms of tsRNA-mediated regulation are not yet well understood [5,19,20,21,22]. Numerous studies have demonstrated that tsRNAs play vital roles in translational regulation through an AGO-independent approach [48]. Specifically, tsRNAs might participate in translational control by interacting with the general translation machinery, or high-order cytoplasmic structures such as polyribosomes, processing bodies, and stress granules. These findings are briefly summarized as follows.

### 4.1. Terminal Oligoguanine (TOG) 5′ tRNA-Halves Displacing Translation Initiation Factors from mRNAs

In mammalian cells, ANG is induced upon stress to generate tRNA-halves which subsequently induce the assembly of stress granules and suppress protein synthesis in an eIF2α-independent manner [30,31,49,50]. Remarkably, two tRNA-halves (5′-tsRNA^Ala^ and 5′-tsRNA^Cys^) whose 5′ ends have four to five guanine residues (“terminal oligoguanine”, or TOG) have significant translation repressive effects [51]. It has been demonstrated that these TOG motifs can form intermolecular RNA G-quadruplexes which displace the translation initiation factor eIF4G/eIF4A from mRNAs and slow down translational initiation [51,52,53,54].

### 4.2. tsRNAs Binding Multi-Synthetase Complex (MSC) to Effect Translation

**Figure 2:**
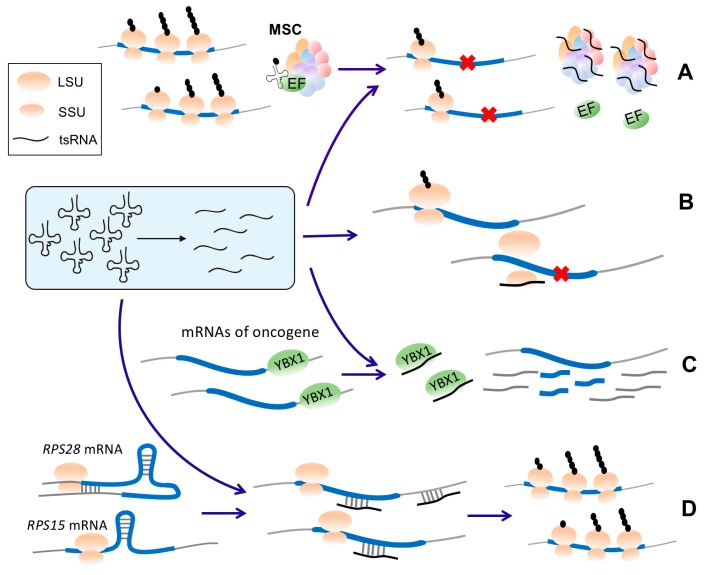
The Argonaute (AGO)-independent translation regulation modes mediated by tsRNAs. (**A**) In human cells, the multi-synthetase complex (MSC) can associate with elongation factors (EFs) to deliver charged tRNAs to the ribosomes. A 5′-tsRNA^Gln^ interferes with this process by interacting with MSC, which impedes protein translation. (**B**) In *Haloferax volcanii*, 5′-tsRNA^Val^ can bind to small ribosomal subunits, which inhibits formation of the translation initiation complex and reduces protein translation under stress conditions. (**C**) Certain oncogenic mRNAs are stabilized if their 3′ UTRs are bound by YBX1. In breast cancer cells, several tsRNAs can competitively bind to YBX1, which destabilizes such oncogenic mRNAs by displacing them from YBX1. (**D**) A 22-nt 3′tsRNA-Leu^CAG^ binds the mRNAs of two ribosomal proteins (*RPS28* and *RPS15*) to unfold their secondary structures and enhance their translation. For *RPS28* mRNA, one target site of 3′tsRNA-Leu^CAG^ is located in the coding sequence (CDS) and the other target site is located in 3′UTR; for *RPS15* mRNA, the tsRNA target site is located in CDS.

During protein biosynthesis in eukaryotes, multiple aminoacyl-tRNA synthetases and accessory factors constitute a multienzyme complex (multi-synthetase complex, or “MSC”), which plays a key role in translational elongation by delivering charged tRNAs to the ribosomes and recycling deacylated tRNAs [55,56,57]. A recent study suggests that human 5′ tsRNA^Gln^ interacts with the MSC, which is associated with global translation repression [58] (Figure 2A). Curiously, it was also discovered that overexpression of the 5′-tsRNA^Gln^ leads to increased translation of ribosomal and poly(A)-binding proteins due to an unknown reason [58].

### 4.3. tsRNAs Competing for Ribosomes

It has been shown that in the halophilic archaeon *Haloferax volcanii*, a stress-induced tsRNA (5′-tsRNA^Val^) can bind to the small ribosomal subunits, which interferes with the translation initiation complex formation of mRNAs and inhibits protein translation [59,60] (Figure 2B).

### 4.4. Competitive Binding of tsRNAs to YBX1 to Destabilize YBX1-Bound mRNAs 

YBX1 is an RNA-binding protein that stabilizes many oncogenic mRNAs by binding their 3′ UTRs. The binding motif recognized by YBX1 is an 8-mer sequence that is called CU-box [11]. The positions 2 and 3 of the CU-box motif are predominantly C and U, respectively. YBX1 can also bind several tsRNAs (derived from tRNA^Glu^, tRNA^Asp^, tRNA^Gly^, and tRNA^Tyr^) that have the CU-box. In breast cancer cells, those tsRNAs are induced and competitively bind to YBX1, which displaces the oncogenic mRNAs from YBX1. As a consequence, the oncogenic transcripts that are displaced from YBX1 by tsRNAs are destabilized, which further suppresses cancer progression [11] (Figure 2C).

### 4.5. tsRNAs Unfolding Secondary Structure of Target mRNAs

Although most tsRNA-mediated regulation is associated with reduced translational efficiency, it has been reported that a 22-nt 3′tsRNA-Leu^CAG^ binds the mRNAs of two ribosomal proteins (*RPS28* and *RPS15*) to unfold their secondary structures and enhance their translation [14,54] (Figure 2D). Interesting, the binding between 3′tsRNA-Leu^CAG^ and the target mRNAs does not require AGO proteins [14]. Additionally, the inhibition of 3′tsRNA-Leu^CAG^ impairs ribosome biogenesis by decreasing the number of 40S ribosomal subunits [14]. Remarkably, the 3′tsRNA-Leu^CAG^ could induce apoptosis in rapidly dividing cells or in a patient-derived orthotopic hepatocellular carcinoma model in mice, highlighting its regulatory roles in cancer [14].

## 5. Regulatory Functions of AGO-Bound tsRNAs

**Figure 3:**
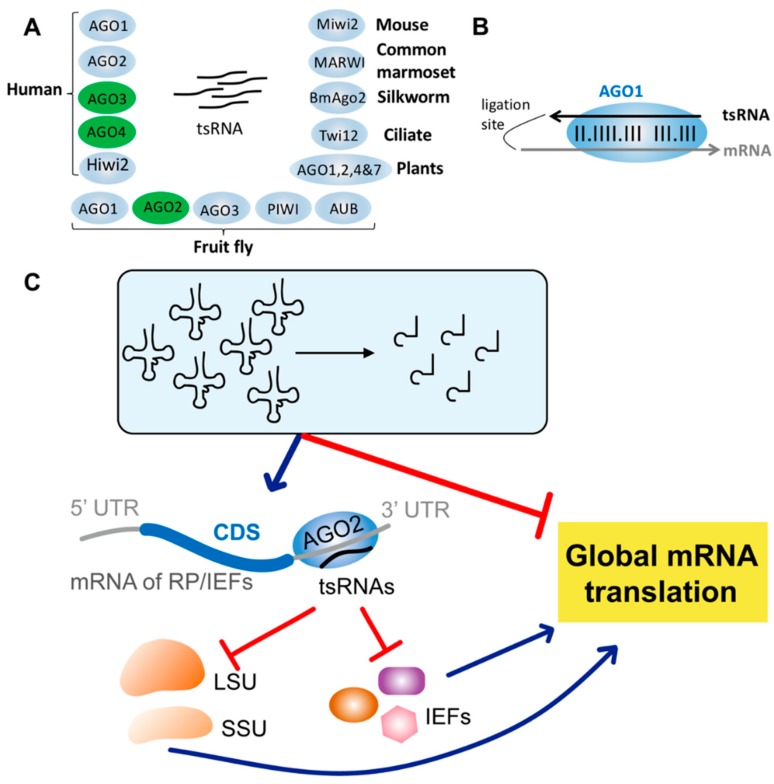
AGO-dependent tsRNA-mediated regulation. (**A**) tsRNAs are bound by AGO proteins in various species. In humans, tsRNAs are preferentially associated with AGO3 and 4 over AGO1 and 2 [68], and tsRNAs are also bound by Hiwi2 [65]. In *Drosophila*, tsRNAs are bound by AGO1, AGO2, AGO3, PIWI, and AUB. In S2 cells, tsRNAs are preferentially bound by AGO2 over AGO1 [23]. tsRNAs are also bound by Miwi2 in mice [65], by MARWI in the common marmoset [66], by BmAgo2 in silkworms [64], by Twi12 in ciliates [61,62], and by AGO1, 2, 4 and 7 in plants [63]. (**B**) The tsRNA-mRNA chimeras that are bound by AGO1 as revealed by human cross-linking, ligation and sequencing of hybrids (CLASH) data. (**C**) *Drosophila* tsRNAs suppress global translation by impeding ribosome biogenesis. *Drosophila* AGO2-bound tsRNAs preferentially inhibit translation of mRNAs of the ribosome proteins (RPs) or translational initiation or elongation factors (IEFs) through a RNAi-like approach, leading to attenuated global translation. Meanwhile, the processing of tRNA into tsRNAs also inhibits global translation since the abundance of tRNAs is reduced.

Although many studies suggest tsRNA-mediated regulation can be exerted through an AGO-independent approach, others have demonstrated that tsRNAs are bound by AGO proteins in a wide range of species, including ciliates [61,62], plants [63], silkworms [64], flies [43], mice [65], the common marmoset [66], and humans [10,67] (Figure 3A). It was found that among human AGO1-4 proteins, tsRNAs are preferentially associated with AGO3 and 4 over AGO1 and 2 in HEK293 cells [68] (Figure 3A). Also, tsRNAs are associated with human Hiwi2, a PIWI paralog in the AGO family that is associated with the piRNA pathway and abundantly expressed in normal and tumor somas [65] (Figure 3A). Furthermore, many studies indicate that the complementarity between tsRNAs and target mRNAs is indispensable for efficient silencing [10,27,32,63,68]. For example, one 22-nt 3′tsRNA-Gly^GCC^ (CU1276) was shown to associate with all four human AGO proteins and repress the target genes in a miRNA-like approach [10]. One ~30-nt 5′tRNA-Glu^CTC^ also suppresses its target genes in Hep-2 cells in a miRNA-like manner, although the pairing is nucleated at the 3′- instead of the 5′-portion of this tsRNA [32]. Remarkably, a re-analysis of the human CLASH (cross-linking, ligation and sequencing of hybrids) data [69] has identified various AGO1-tsRNA-mRNA chimeras (Figure 3B), which suggests that tsRNAs regulate targets via an RNAi-like manner [27]. Moreover, in *Tetrahymena thermophile*, the Piwi protein Twi12 binds 3′-tsRNAs to activate Xrn2 to induce the processing of cellular rRNAs [61] (Figure 3A). Altogether, these studies demonstrate that in many circumstances tsRNAs are bound with AGO proteins to exert the regulatory functions.

## 6. *Drosophila* tsRNAs Inhibiting Global Translation by Impeding Ribosome Biogenesis

Among the five AGO proteins in *Drosophila*, AGO1 and AGO2 primarily bind miRNAs and endogenous siRNAs, respectively; AGO3, PIWI, and AUB, which are mainly expressed in germline cells, primarily bind piRNAs [2,70] (Figure 3A). By analyzing the small RNAs that were associated with different AGO proteins (IP-Seq), we previously found that the short tsRNAs that have similar lengths as miRNAs or siRNAs (20–22 nt) are preferentially bound by AGO2 over AGO1 in *Drosophila* S2 cells, and the long tsRNAs that have similar lengths as piRNAs (24–29 nt) tend to be bound by AGO3, AUB and PIWI in the ovaries. These results suggest that tsRNAs might exert a regulatory function through an RNAi-like approach. To further probe the function of the tsRNAs, we selected 12 tsRNAs (most of them are bound by AGO2 in the IP-Seq data) and transfected each individual single-stranded tsRNA mimic into S2 cells. The polysome profiling analyses indicated the majority of tsRNAs had profound inhibitory effects on global mRNA translation. Interestingly, our mRNA-Seq and Ribo-Seq [71,72] experiments after cellular transfection of three tsRNAs suggest these tsRNAs recognize target mRNAs through conserved antisense matching and suppress translation rather than degrade mRNAs of the target genes [23].

The target prediction results suggested that AGO2-bound tsRNAs preferentially suppress translation of the key components of the general translation machinery such as ribosome proteins (RPs) and translational initiation or elongation factors (IEFs). We also found the expression profiles of the AGO2-bound tsRNAs reprogrammed under cellular stress to modulate translation of the RPs or IEFs. Moreover, we showed that tsRNA-mediated repression is dependent on AGO2 in *Drosophila*. First, we found the translational efficiencies of tsRNA targets are upregulated after AGO2 knock-down in S2 cells, and this pattern is especially strong for RPs or IEFs. Second, we found the targets of the upregulated tsRNAs are downregulated in translation during starvation. However, this pattern disappeared when we knocked down AGO2. Our results suggested *Drosophila* AGO2-bound tsRNAs inhibit global translation by preferentially suppressing translation of RPs or IEFs through an RNAi-like approach (Figure 3C). Our results also suggested that tsRNA-mediated regulation may be crucial for energy homeostasis and metabolic adaptation in cellular systems [23].

Why do tsRNAs preferentially target mRNAs of RPs or IEFs? We found *Drosophila* AGO2 preferentially bound “AAG” containing 7-mer motifs that are significantly enriched in the mRNAs of RPs or IEFs. Therefore, we speculate that the antisense pairing between tsRNAs and mRNAs of RPs or IEFs might be facilitated by the tethering effects of AGO2. It is possible that the preferential pairing between AGO2-bound tsRNAs and mRNAs of RPs or IEFs are due to long-term co-evolution between their sequences. 

## 7. Concluding Remarks and Future Perspectives: Taking *Drosophila* tsRNAs as an Example

The tsRNA-mediated regulation adds another layer of regulatory complexity. Understanding the fundamental biology of tsRNAs remains the subject of intense interest and deserves further systematic studies. However, significant gaps remain in our understanding of the biogenesis, molecular mechanisms and evolutionary principles of tsRNA-mediated gene regulation. Specifically, taking tsRNAs in *Drosophila* as an example, the following issues remain to be addressed in the near future.

### 7.1. The Biogenesis of Drosophila tsRNAs

We previously found that in *Drosophila*, the short tsRNAs that have similar lengths as miRNAs or siRNAs (20–22 nt) are preferentially bound by AGO2 over AGO1 in *Drosophila* S2 cells, and the long tsRNAs that have similar lengths as piRNAs (24–29 nt) tend to be bound by AGO3, AUB and PIWI in the ovaries [23]. However, the genes and pathways crucial for tsRNA biogenesis in the soma and germlines are still poorly understood. Although ANG and Rny1 are involved in the biogenesis of tRNA-halves in vertebrates and yeasts respectively, ANG does not exist in *Drosophila*, and whether the ortholog of Rny1 in *Drosophila* participates in tsRNA biogenesis is still questionable. Moreover, whether the key players participating in the miRNAs or siRNAs biogenesis pathways such as *Dcr-1* and *r2d2* influence tsRNA generation is not well understood at this moment [23,27]. Interestingly, it has been nicely demonstrated that the biogenesis of tsRNAs is dependent on key factors in the piRNA pathway such as Armi, Zuc, and dGasz in the *Drosophila* KC167 cell line [73]. Altogether, these studies suggest that the biogenesis of tsRNAs might be different in the soma and germlines of *Drosophila*, and future studies are required to better understand the detailed mechanisms underlying the biogenesis of tsRNAs.

### 7.2. The Molecular Mechanism by which Drosophila tsRNAs Inhibit Translation of the Targets

Our results suggest that the AGO2-bound tsRNAs recognize target mRNAs through conserved antisense matching and suppress translation rather than degrade mRNAs of the target genes in *Drosophila* [23]. However, the molecular mechanisms underlying the tsRNA-mediated translational suppression are not well understood at this moment. Functional genomics approaches such as CLEAR (covalent ligation of endogenous Argonaute-bound RNAs)-CLIP [74] might help identify the in vivo tsRNA-target chimera and help decipher the molecular mechanism by which *Drosophila* tsRNAs inhibit translation of the targets.

### 7.3. The Function of piRNA-like tsRNAs in Drosophila Germlines

Recent studies indicate that mammalian tsRNA are crucial for sperm maturation and fertilization in mammals [6,17], or the intergenerational inheritance of an acquired metabolic disorder [18,75]. In the ovaries of *Drosophila*, the tsRNAs are associated with AGO3, AUB, and PIWI and have similar length distributions (23–29 nt) as piRNAs [23]. We previously found the translational efficiency of the top target mRNAs of the AUB-bound tsRNAs is significantly lower than that of the non-targets in the mature oocytes but not in the activated eggs [23]. Hence, it is possible that the piRNA-like tsRNAs might play important roles in translational control during the development of the *Drosophila* germlines and early embryos. However, further studies are required for a better understanding of the detailed mechanisms in this process.

### 7.4. The role of RNA Modifications on Biogenesis and Function of tRNAs.

There are extensive post-transcriptional modifications in tRNAs [76,77,78,79]. Several studies have demonstrated how tRNA modifications might affect the biogenesis and functions of tsRNAs. A considerable number of TOG-containing tsRNAs in human embryonic stem cells (hESCs) are centered at 18 nt, and these endogenous 5′ tsRNAs are termed mini TOGs (mTOGs) [80]. The uridine residues at position 8 (U8) of the mTOGs that harbor a specific Ψ-consensus motif could be highly modified as Ψ by the PUS7 Ψ synthase in hESCs. It has been shown that that Ψ could strongly promote the association between mTOGs and polyadenylate-binding protein 1 (PABPC1), which regulates mRNA translation by inhibiting PABPC1 recruitment to eIF4F and controls cell fate determination in stem cells [80]. Also, a previous study suggested the methylation of tRNA mediated by Dnmt2 (DNA methyltransferase 2) could inhibit stress-induced ribonuclease cleavage of tRNAs in *Drosophila*, which suggests that Dnmt2-mediated tRNA methylation plays a regulatory role in tsRNA biogenesis under stress [81]. NSUN2 is the cytosine-5 RNA methylase that methylates the vast majority of tRNAs. NSUN2-mediated tRNA methylation could protect tRNAs from ANG cleavage in mice and humans [82,83]. The loss of NSUN2-mediated cytosine-5 tRNA methylation could lead to elevated levels of tsRNAs that are generated by ANG cleavage, which further impairs mammalian brain development [82]. However, given the high diversity of tRNA modifications across different tissues, developmental stages and species, intense efforts are needed to decipher the relationship between tRNA modification and the biogenesis and function of tsRNAs in future studies.
